# The profile of young people seeking chiropractic care: a secondary analysis of data

**DOI:** 10.1186/s12998-026-00677-5

**Published:** 2026-09-15

**Authors:** Melissa Neave, Michael Swain, Aron Downie, Katie de Luca, Sheilah Hogg-Johnson, Silvano Mior, Simon French

**Affiliations:** 1 Department of Chiropractic, Spinal Pain Research Centre, Macquarie University, Sydney, NSW Australia; 2 Chiropractic Course, School of Health, Medical and Applied Sciences, CQUniversity, Brisbane, QLD Australia; 3 Department of Research and Innovation, Canadian Memorial Chiropractic College, Toronto, Canada; 4 Institute for Disability and Rehabilitation Research, Ontario Tech University, Toronto, Canada

**Keywords:** Health services, Chiropractic, Musculoskeletal disorders, Children, Paediatric, Young people, Manipulation

## Abstract

**Background:**

Chiropractic care for children has increased significantly in recent years and is now a commonly utilised healthcare option. Despite this, chiropractic’s role in paediatric healthcare remains contentious. We performed a secondary analysis of two cross-sectional studies of chiropractic practice in Australia and Canada to describe the characteristics of paediatric patients presenting to chiropractors and to examine the reasons for seeking care, diagnosed conditions, and treatments provided across three age strata.

**Methods:**

The Chiropractic Observation and Analysis Study (COAST) and the Ontario Chiropractic Observation and Analysis Study (O-COAST) are cross sectional observational studies conducted in Victoria, Australia (2010–2012) and Ontario, Canada (2014–2015), designed to provide a better understanding of chiropractic practice and trends. Patient encounters of individuals under 18 years of age were identified and extracted from respective datasets. Paediatric patients were categorised into three age strata based on neurodevelopmental and musculoskeletal maturation stages: 0–23 months, 2–9 years and 10–17 years. Descriptive data regarding patient characteristics, reason for encounter, and treatment received from the chiropractor were tabulated for analysis.

**Results:**

Of all 670 paediatric encounters, 20% were for 0–23 months, 33% were for 2–9 years and 47% for 10–17 years. “Health maintenance” was the most common reason for encounter in each age stratum. Non-musculoskeletal problems were most common in the 0–23 months stratum. Chiropractors most frequently diagnosed “back problems” in each age stratum. Manual adjustment was the most common treatment delivered overall.

**Conclusion:**

We described the reasons paediatric patients seek chiropractic care, the conditions diagnosed by chiropractors, and the techniques used across three age groups. An incongruence was identified between patients’ reasons for seeking care and chiropractors’ diagnoses. Understanding the characteristics of paediatric patient groups and the patterns of presentation and treatment will aid in designing relevant research, to inform clinical practice and teaching programs, and implement evidence-based health policies.

**Supplementary Information:**

The online version contains supplementary material available at https://doi.org/10.1186/s12998-026-00677-5.

## Introduction

Musculoskeletal conditions are a significant burden on the health of children and adolescents globally [1, 2]. The consequences of these conditions extend beyond functional impediments to include high societal and economic costs in the form of increased healthcare utilisation by young people and lost productivity of parents and caregivers [3]. It has been estimated that musculoskeletal conditions are responsible for up to 8% of paediatric presentations to general medical practice in primary care [4–6], comprising mostly lower limb, neck, and back complaints [4–6]. These complaints commonly coexist, persist, or reoccur over time [7–10], which adds to healthcare burden and costs [11]. A recent call to action by the Paediatric Global Musculoskeletal Task Force, highlights the need to increase healthcare workforce capacity to care for young people with musculoskeletal complaints [12], suggesting the importance of early intervention and appropriate management strategies to ameliorate the risk of chronic musculoskeletal complaints which progress into adulthood [13–17]. Considering the unmet workforce need aligns with the core objectives of chiropractic care, it is pertinent to examine the role of chiropractors in addressing musculoskeletal complaints in young people.

Chiropractic is a regulated primary healthcare profession in many parts of the world, including Australia and Canada, and is predominately concerned with the diagnosis, treatment and prevention of musculoskeletal disorders and the effects of these disorders on general health [18, 19]. While patients of all ages seek chiropractic care, the annual median utilisation rate of chiropractic care for individuals less than 18 years of age in high income countries is estimated to be 8% [20]. Musculoskeletal complaints are the most common reason for paediatric patients to seek chiropractic care, but some studies report non-musculoskeletal conditions among the presentations for infants and young children. For example, excessive crying, gastrointestinal complaints and disturbed sleep may be the presenting complaint to chiropractors for infants and young children, while low back pain and headache are often the presenting complaint for older children and adolescents [21–26]. Recent evidence-based consensus statements have been developed to guide chiropractic care of children and adolescents [27, 28]. However, there is little research that details the reasons young people seek chiropractic care, nor the nature of the care provided.

Knowledge of chiropractic care of paediatric populations is currently derived from retrospective studies that rely on chiropractors’ recall, resulting in a lack of robust health service data [23–25, 29–32]. The paucity of reliable data extends to several key areas of paediatric chiropractic practice including: the reasons paediatric patients seek chiropractic care, the specific health conditions that chiropractors treat for their paediatric patients, and the treatment modalities employed by chiropractors for patients across paediatric age groups. This knowledge gap hinders the ability to assess the role of chiropractic care in paediatric health service provision and impedes evidence-based decision-making for practitioners and policymakers [33].

The Chiropractic Observation and Analysis STudy (COAST) and the Ontario Chiropractic Observation and Analysis STudy (O-COAST) collected data from nearly 8,000 chiropractic patient encounters in Australia and Canada [34, 35]. The purpose of this current study was to conduct a secondary analysis of COAST and O-COAST data to describe the characteristics of young patients seeking chiropractic care, the reasons for encounter, the diagnoses made, and the type of treatments provided by chiropractors.

## Methods

### Study design, setting, participants

We conducted a secondary analysis of COAST and O-COAST data [34, 35]. In brief, these cross-sectional studies, which employed the same study design, recruited 52 chiropractors from Victoria, Australia (33% response rate) and 39 chiropractors from Ontario, Canada (35% response rate). Chiropractors in both studies recorded details on a maximum of 100 consecutive patient encounters over a four-week period between December 2010 and September 2012 for COAST, and between August 2014 and November 2015 for O-COAST. In both studies, chiropractors were provided with standardised paper encounter forms with free-text or check-box options to record patient characteristics (age, sex, height, weight, co-morbidities), up to three reasons for the encounter (RFE), up to three diagnoses, and techniques and treatment provided during the encounter. Referrals to and from other healthcare providers, any imaging ordered and payment method for the visit were also recorded. Sociodemographic and clinical practice data from the participating chiropractors were also collected. Diagnoses and RFE were categorised by trained coders using the International Classification of Primary Care, 2nd edition (ICPC-2) PLUS general practice terminology and the ICPC-2 PLUS Chiro terminology which was developed for chiropractic specific RFE and diagnoses [36].

### Ethical approval

for COAST was obtained from the University of Melbourne Human Research Ethics Committee (HREC: 0931651) and for O-COAST from the Canadian Memorial Chiropractic College (REB: 1404 × 03) and Queen’s University (REB: 6012853) ethics boards. This study conforms to the appropriate reporting guidelines for observational studies (cross-sectional studies) in accordance with the STROBE (Strengthening the Reporting of OBservational studies in Epidemiology) guidelines [37]. Ethical approval for this secondary analysis was approved by Macquarie University Human Research Ethics Committee (HREC: 520251896065922).

### Data sources, measurements, and transformations

For this secondary analysis, chiropractic RFE data from the COAST and O-COAST studies were restricted to patients whose age was recorded as 17 years of age or younger. Chiropractic encounter data were categorised into three age-groups based on clinically appropriate age ranges related to neurodevelopmental and musculoskeletal maturation stages [38]. Moreover, the age-groups were chosen to ensure adequate numbers in each stratum to allow meaningful interpretation of the data, which included: infant: 0–23 months; children: 2–9 years old and adolescent: 10–17 years old.

### Reasons for encounter (RFE) and diagnoses

The ICPC-2 PLUS coded RFE and diagnoses were collapsed into 31 categories to combine specific presentations and diagnoses into regional (e.g. knee pain and ankle pain into “lower extremity problem”) or condition specific (e.g. cough, cold symptoms, into “respiratory”) categories to allow for meaningful comparison between age strata. As the aetiology of colic is contentious [39], RFE and diagnoses labelled “colic” and descriptions of unsettled and irritable behaviour were categorised as “Irritable infant”. All RFEs and diagnoses described as abdominal pain, upset stomach and regurgitation, etc., were categorised as “gastrointestinal”. Terms such as “wellbeing”, “wellness” and “check-up” were deemed synonymous and were grouped as Health Maintenance.

### Treatment modalities

Chiropractic treatment modalities were categorised into nine categories based on techniques recorded on the data collection forms. For this analysis the “other” category included chiropractor described visceral and cranial based techniques and chiropractic systems of analysis. “Activator” refers to any treatment that described the use of a mechanically assisted adjusting tool (e.g. activator, arthrostim). “Modalities” were categorised as an electrical or thermal treatment.

### Statistical analysis

From each of the COAST and O-COAST datasets, encounters of patients 17 years and younger were identified using date of birth and date of encounter. Participant records were excluded if combinations of height, weight and occupation were illogical given calculated age. Pooling of the COAST and O-COAST datasets was undertaken to increase sample size and was considered appropriate given the comparable study designs, data collection procedures, and measurement methods employed across the two studies [40].

Descriptive statistics were used to summarise patient and encounter characteristics. Patient characteristics were compared between age groups (0–23 months, 2–9 years, 10–17 years) using χ^2^ tests, accounting for the clustering of patients within chiropractors by using survey estimator procedures with chiropractor as the primary sampling unit. Encounters, rather than individual RFE, diagnosis, or technique counts, served as the denominator for all reported rates. Diagnoses, RFEs and techniques were expressed as rates per 100 encounters with 95% Confidence Intervals (95%CI). Confidence intervals for rates employed survey estimator procedures to account for clustering within chiropractors. All analyses were conducted using Stata version 10 (StataCorp. 2007. Stata Statistical Software: Release 10. College Station, TX: StataCorp LP) and SAS software (Copyright © 2018 SAS Institute Inc.).

## Results

Of 7,987 clinical encounters (4,464 from COAST and 3,523 from O-COAST) there were 670 paediatric encounters from 605 discrete paediatric patients (8.4% CI 7.8%-9.0%). Three encounters were excluded from the COAST data and 13 from the O-COAST data due to doubt regarding date of birth and corresponding height, weight, and occupation characteristics.

### Chiropractors

Of the 91 chiropractors who participated in the COAST and O-COAST studies, 79 (87%) reported a paediatric encounter representing 92% (48/52) of participating Australian and 79% (31/39) of Canadian chiropractors, respectively. Of participating chiropractors who reported a paediatric encounter, 70% were male, with a mean age of 43.4 years and had been in practice a mean 17.6 years. Three chiropractors reported holding (unspecified) paediatric qualifications. The median number of paediatric encounters per chiropractor during their 100 encounters was 5 (IQR 2–10). Table 1 details the characteristics of participating chiropractors from both studies.

### Paediatric patient demographics and patient encounters

This analysis revealed 47% of paediatric patients were aged between 10 and 17 years old, 33% were aged 2–10 years old and 20% were aged less than two years (Table 2). Ten percent of paediatric patients were new patients to the chiropractors during the study period. The majority of children (92%) attended one encounter during the study period, with 2% of patients attending three or more chiropractic encounters during the study period. Chiropractors saw more male paediatric patients than females (56% male). One or more co-morbidities were recorded in 10% of encounters, which included conditions such as food allergies, asthma, diabetes and cystic fibrosis. Imaging was requested in 11 (2%) encounters. The main referral source in each age group was “other patients” (60%) followed by “family and friends” (5%). Nurses, midwives and lactation coaches were the second largest referral source in those aged under two (14%, 5% overall). In total, young people were referred by a physician in 13 encounters (2%), of which 12 were in the oldest age group (4% of the stratum).

### Reasons for encounter

Table 3 reports the ten most frequent RFE in each age stratum. “Health maintenance” was the leading RFE across all three groups: 61.4, 58.2, and 34.7 per 100 encounters for infants (0–23 months), children (2–9 years), and adolescents (10–17 years), respectively.

A spectrum of musculoskeletal and non-musculoskeletal RFE were reported across age groups. In infants, sleep problems at 19.3 and feeding issues at 10.7 per 100 encounters, were unique to this group. In children, neck at 8.5 and back problems at 5.6 per 100 encounters, were the most common RFEs after health maintenance, followed by psychological complaints at 4.7, ear problems, and respiratory complaints (both 4.2 per 100 encounters). In adolescents, back problems at 34.1, lower extremity at 14.8, and neck problems at 11.7 per 100 encounters, were the most common musculoskeletal RFEs after health maintenance, with headache at 6.9 per 100 encounters, the most frequently reported non-musculoskeletal RFE.

**Table 1:** Characteristics of 79 participating chiropractors providing care for paediatric patients in COAST and O-COAST

Chiropractors	Estimates
Female (%)	26 (32.9%)
Mean age in years (range, SD)	43.4 (24–71, 10.4)
Mean years in practice (range, SD)	16.6 (1–45, 9.6)
Mean years since graduation (range, SD)	17.6 (1–45, 9.7)
Country of chiropractic education	
Australia	40 (50.6%)
Canada	29 (36.7%)
Other (USA/NZ/UK)	10 (12.7%)
Postgraduation qualifications (%)	22 (28.2%)
Paediatric qualifications (%)	3 (3.8%)
Median number of paediatric encounters per chiropractor* (range, IQR)	5 (1–87, 2–10)
0–23 months	0 (0–55; 0–1)
2–9 years	1 (0–28; 0–3)
10–17 years	3 (0–16; 2–6)
Practice	
Mean number patient visits per week (range, SD)	95.8 (8-250, 62.7)
Practice type *(multiple responses possible)* (%)	
General/family	76 (96.2%)
Sports/rehab	9 (11.4%)
Paediatric	3 (3.8%)
Wellness/lifestyle	1 (1.3%)

* for chiropractors who recorded any paediatric encounter

**Table 2:** Characteristics of paediatric patients seeking chiropractic care in COAST and O-COAST (*n* = 605)

	Total -All Ages (0–17 years)	0–23 months	2–9 years	10–17 years	Comparison between age groups
Number of encounters	670	139	214	317	
Number of patients	605	123	200	282	
Encounters per patient 1 encounter 2 encounters 3 + encounters	554 (91.6%) 40 (6.6%) 11 (1.8%)	109 (88.6%) 11 (8.9%) 3 (2.4%)	188 (94.0%) 11 (5.5%) 1 (0.5%)	257 (91.1%) 18 (6.4%) 7 (2.5%)	χ^2^ = 5.6, df = 4, *p* = 0.2
New patient	52/524 (9.9%) (81 missing)	16/109 (14.7%) (14 missing)	14/170 (8.2%) (30 missing)	22/245 (9.0%) (37 missing)	χ^2^ = 4.9, df = 2, *p* = 0.09
Male Female	333 (56.2%) 260 (43.8%) (12 missing)	73 (60.8%) 47 (39.2%) (3 missing)	110 (56.1%) 86 (43.9%) (4 missing)	150 (54.1%) 127 (45.9%) (5 missing)	χ^2^ = 1.3, df = 2, *p* = 0.5
Referred by/from Other patient Family / Friend Nurse /Midwife /Lactation coach Other Chiro Physician Other provider Other	361 (59.7%) 32 (5.3%) 27 (4.5%) 21 (3.5%) 13 (2.2%) 11 (1.8%) 8 (1.3%)	76 (61.8%) 8 (6.5%) 17 (13.8%) 6 (4.9%) 0 (0.0%) 4 (3.3%) 4 (3.3%)	120(60.0%) 10 (5.0%) 9 (4.5%) 10 (5.0%) 1 (0.5%) 2 (1.0%) 3 (1.5%)	165 (58.5%) 14 (5.0%) 1 (0.4%) 5 (1.8%) 12 (4.3%) 5 (1.8%) 1 (0.4%)	χ^2^ = 0.2, df = 2, *p* = 0.9 χ^2^ = 0.5, df = 2, *p* = 0.8 ^ χ^2^ = 5.2, df = 2, *p* = 0.07 ^ χ^2^ = 3.6, df = 2, *p* = 0.2 χ^2^ = 2.7, df = 2, *p* = 0.3
≥ 1 comorbidity	64 (10.6%)	10 (8.1%)	19 (9.5%)	35 (12.4%)	χ^2^ = 1.4, df = 2, *p* = 0.5
Referred for imaging	11 (1.8%)	2 (1.6%)	2 (1.0%)	7 (2.5%)	χ^2^ = 1.0, df = 2, *p* = 0.6

^ statistic cannot be computed due to small cells and distribution across chiropractors

**Table 3:** Most common reasons for encounter recorded by chiropractors, ranked by frequency and stratified by age

0–23 months			2–9 years			10–17 years		
Rank	Reason for Encounter Group	Rate/100 (95% CI)	Rank	Reason for Encounter Group	Rate/100 (95% CI)	Rank	Reason for Encounter Group	Rate/100 (95% CI)
1	Health maintenance	61.4 (44.8–84.1)	1	Health maintenance	58.2 (47.3–71.7)	1	Health maintenance	34.7 (28.8–41.8)
2	Sleep problem	19.3 (10.2–36.4)	2	Neck problem	8.5 (4.6–15.6)	2	Back problem	34.1 (26.9–43.1)
3	Gastrointestinal	12.1 (7.4–20.0)	3	Back problem	5.6 (2.6–12.0)	3	Lower extremity problem	14.8 (9.3–23.6)
4	Feeding issues	10.7 (5.8–19.9)	4	Psychological	4.7 (2.0–11.2)	4	Neck problem	11.7 (7.7–17.7)
5	Irritable infant	10.0 (5.8–17.3)	5	Ear	4.2 (2.4–7.5)	5	Headache	6.9 (4.3–11.3)
6	Neck problem	5.7 (3.0–10.9)	5	Respiratory	4.2 (2.1–8.5)	6	Upper extremity problem	6.6 (4.0–11.0)
6	Posture	5.7 (1.5–21.5)	7	Upper extremity problem	3.3 (1.4–7.5)	7	Psychological	3.8 (1.8–8.1)
8	Other non-specific	4.3 (2.1–8.8)	8	Posture	2.8 (1.3–6.2)	8	Other / non-specific MSK	3.5 (1.9–6.4)
9	Trauma/Injury	3.6 (1.3–9.5)	8	Urinary	2.8 (1.2–6.7)	9	Concussion	2.5 (0.5–13.6)
9	Upper extremity problem	3.6 (0.9–13.5)	10	Headache	2.3 (1.0–5.7)	9	Other non-specific	2.5 (1.3–5.0)
			10	Trauma/injury	2.3 (0.8–6.6)	9	Posture	2.5 (1.1–6.0)
			10	Other non-specific	2.3 (1.0–5.5)			
			10	Speech	2.3 (0.5–11.4)			
			10	Lower extremity problem	2.3 (1.1–5.2)			

Note. Rank reflects descending order of reported rate within each age group. Where rates were equal, reasons for encounter were assigned the same rank, and the subsequent rank was incremented accordingly (CI = confidence interval; MSK = musculoskeletal

**Table 4:** Most common diagnoses recorded by chiropractors, ranked by frequency and stratified by age

0–23 months			2–9 years			10–17 years		
Rank	Diagnostic Group	Rate/100 (95% CI)	Rank	Diagnostic Group	Rate/100 (95% CI)	Rank	Diagnostic Group	Rate/100 (95% CI)
1	Back problem	38.6 (20.7–71.8)	1	Back problem	62.0 (43.6–88.0)	1	Back problem	66.6 (53.8–82.4)
2	Health maintenance	24.3 (11.7–50.3)	2	Neck problem	15.5 (7.5–31.9)	2	Other / non-specific MSK	15.5 (10.1–23.6)
3	Neck problem	10.7 (4.7–24.4)	3	Health maintenance	12.7 (6.6–24.4)	3	Neck problem	13.2 (8.8–20.0)
4	Other / non-specific MSK	10.0 (3.8–26.5)	4	Other / non-specific MSK	9.4 (4.9–18.1)	4	Lower extremity problem	10.7 (6.8–17.0)
4	Sleep problem	10.0 (3.1–32.4)	5	Lower extremity problem	3.3 (1.3–8.4)	5	Health maintenance	6.6 (3.1–14.4)
6	Gastrointestinal	7.9 (4.2–14.8)	6	Neurological	2.8 (1.0–8.3)	6	Upper extremity problem	5.0 (2.9–8.7)
7	Irritable infant	6.4 (3.0–13.8)	6	Respiratory	2.8 (1.3–6.0)	7	Headache	4.7 (2.8–8.1)
8	Feeding problem	5.7 (1.5–21.5)	8	Ear	2.3 (1.0–5.6)	8	Concussion	2.5 (0.6–10.9)
9	Upper extremity problem	5.0 (1.3–18.8)	8	Gastrointestinal	2.3 (0.6–9.9)	9	Neurological	1.9 (0.7–5.4)
10	Lower extremity problem	4.3 (1.2–15.7)	10	Headache	1.9 (0.7–5.4)	9	Other / non-specific	1.9 (0.6–5.7)
10	Posture	4.3 (1.1–16.1)	10	Posture	1.9 (0.7–5.4)			
			10	Psychological	1.9 (0.4–8.9)			
			10	Urinary	1.9 (0.5–7.4)			

Note. Rank reflects descending order of reported rate within each age group. Where rates were equal, diagnostic groups were assigned the same rank, and the subsequent rank was incremented accordingly. CI = confidence interval; MSK = musculoskeletal

**Table 5:** Most common techniques used during paediatric chiropractic encounters, ranked by frequency and stratified by age

0–23 months			2–9 years			10–17 years		
Rank	Treatment	Rate/100 (95% CI)	Rank	Treatment	Rate/100 (95% CI)	Rank	Treatment	Rate/100 (95% CI)
1	Manual adjustment	51.4 (28.5–92.8)	1	Manual adjustment	63.4 (48.1–83.5)	1	Manual adjustment	87.7 (74.0–103.9)
2	Activator/Instrument	42.9 (16.4–112.1)	2	Activator/Instrument	62.0 (42.0–91.4)	2	Soft tissue therapy	66.6 (50.9–87.1)
3	Other	42.1 (26.3–67.6)	3	Soft tissue therapy	46.0 (29.3–72.2)	3	Activator/Instrument	39.1 (27.2–56.2)
4	Soft tissue therapy	28.6 (11.5–71.2)	4	Other	27.7 (15.1–50.9)	4	Drop table	21.1 (13.4–33.2)
5	Mobilisation	13.6 (4.3–43.2)	5	Mobilisation	10.3 (5.6–18.9)	5	Blocks	18.6 (9.0–38.5)
6	Drop table	12.9 (7.0–23.6)	6	Blocks	9.4 (4.0–22.1)	6	Other	12.6 (6.9–22.9)
7	Therapeutic exercise	2.1 (0.6–7.4)	7	Drop table	7.0 (3.1–15.8)	7	Mobilisation	11.7 (7.2–18.9)
8	Blocks	1.4 (0.2–11.5)	8	Therapeutic exercise	2.3 (0.5–11.9)	8	Modalities	4.4 (2.1–9.4)
			9	Modalities	1.9 (0.5–6.6)	9	Acupuncture	4.1 (0.8–20.9)
						10	Therapeutic exercise	1.9 (0.6–6.4)
						11	Flexion distraction	0.9 (0.2–4.3)

Note. Rank reflects descending order of reported rate within each age group. Blank cells indicate no treatment recorded at that rank within the corresponding age group. CI = confidence interval

### Diagnosis

A total of 906 diagnoses were recorded for 670 paediatric encounters. Across each age strata, “Back Problem” which included low back, pelvis and thoracic complaints, was the most common diagnosis made by chiropractors at 38.6, 62.0 and 66.6 per 100 encounters for infants (0–23 months), children (2–9 years), and adolescents (10–17 years), respectively. Table 4 reports the rate of diagnoses per 100 encounters, as recorded by the chiropractor, for each age group.

### Chiropractic treatment provided

Manual adjustment was reported as the most common technique used by the chiropractors across all ages: 51, 63 and 88 per 100 encounters for infants (0–23 months), children (2–9 years), and adolescents (10–17 years), respectively. In all age groups, multiple techniques were commonly reported per encounter. Table 5 reports the rate of technique use per encounter, for each age group.

## Discussion

This study provides information from a secondary analysis of data on chiropractic health services use by young patients in Australia and Canada. Patients younger than 18 years of age comprised 8.4% of chiropractic encounters, and approximately half of these encounters were for adolescents. There was some disparity between parent or patient reported RFE and the diagnosis made by chiropractors. For example, health maintenance was the most common reason reported by parents or patients for seeking chiropractic care across all age-groups followed by musculoskeletal issues, while a back problem was the most common diagnosis made by chiropractors. This disparity may in part reflect the structure of the data collection form, which requested a working diagnosis for each encounter, potentially prompting chiropractors to record a specific problem, such as a back problem, even where the parent- or patient-reported reason for encounter was health maintenance. Manual adjustment was the most common treatment used by chiropractors during paediatric encounters, albeit most chiropractors reported a multi-modal approach, using more than one treatment during encounters. In Australia, chiropractors are currently restricted by the Chiropractic Board of Australia from manipulating children under 2 years of age [41]. It should be noted that the data collection for COAST study occurred before the interim ban on spinal manipulation for infants was implemented.

Our study provides new insights into the characteristics of chiropractic care for young people. Unlike similar investigations into chiropractic practice characteristics in the United States and Scandinavia, where babies less than 12 months old were the largest age group of paediatric patients presenting to chiropractors,^21, 22, 42^ our study of Australian and Canadian chiropractors most frequently encountered adolescent patients. This finding may be explained by our sample of predominately general practice chiropractors, few of which reported any further paediatric specific training. The majority of young patients were seen once during the study period, and referred by another patient of the practice, which may have been a parent [43]. Similar to Danish and Norwegian studies, general practitioners were more likely to refer adolescents, and healthcare workers such as midwives and lactation consultants more likely to refer infants for chiropractic care [21, 22]. The COAST and O-COAST studies did not collect data on the specific conditions for which healthcare professionals recommended chiropractic treatment. Additionally, there is a general lack of comprehensive information regarding the types of conditions that prompt general practitioners and other allied health professionals to refer patients to chiropractors [44]. However, considering 10% of encounters with young patients recorded having one or more co-morbidity, such as diabetes or asthma, understanding referral pathways and interprofessional collaboration among healthcare providers, would likely enhance patient outcomes. This gap highlights the need for further research not only to clarify referral patterns but also to investigate the mechanisms and potential associations between common comorbidities, particularly asthma, and the care these younger patients receive.

A limiting factor in adequate interprofessional communication may lie in the use of profession-specific terminology used to describe RFE. The concept of preventive care is common in the chiropractic management of young patients [21–24, 32]. In this type of clinical encounter there is often no symptom or complaint reported as the RFE. These presentations were labelled with various terms such as “wellness care” or “check-up” and were grouped and categorised as Health Maintenance using the chiropractic specific coding tool developed for the COAST studies. This category was the most common RFE across all age strata, particularly in the 0–23 month olds and 2–9 year olds. This observed difference may be due to the age of the patient and align more with parental reported RFE or perhaps due to the more accepted attitude of periodic health screening common in the younger ages [45]. Health maintenance was also frequently reported as a diagnosis, in all age strata. How a diagnosis of “health maintenance” alters treatment delivery compared to a diagnosis of a back or a neck problem is unclear and warrants further study.

While musculoskeletal conditions were common in older age groups, complaints associated with sleeping, feeding and temperament featured more prominently in young patients aged 0–23 months. Despite limited research evidence to support the use of spinal manipulation in the treatment of non-musculoskeletal conditions [46], our findings are consistent with other studies worldwide that report many parents continue to seek chiropractic care for their children’s non-musculoskeletal childhood conditions [21–26, 42, 47]. Given the prevalence of non-musculoskeletal conditions as a reason for seeking chiropractic care, more research is needed to gain a detailed understanding of the clinical characteristics of these conditions among presenting patients. This includes investigating the components of care provided and whether they are directed towards the infant or the parents, targeting psychological, social, and/or biomechanical mechanisms [48].

We found the use of manual adjusting was ubiquitous across all strata. Manual therapy was a commonly used treatment for adolescents where back problems and non-specific musculoskeletal conditions were more commonly diagnosed. This may be explained by skeletal maturity and musculature of adolescents compared to younger groups, or perhaps the more common presentation of back pain that clinicians were familiar in treating similar to their adult patients. Due to the scarcity of dedicated research and guidelines for child and adolescent spinal pain, chiropractors are left with limited options, often necessitating the use of adult-oriented approaches in the care of young people [13]. A recent review by Yu et al.^17^ supports the use of spinal manipulation for adolescent back pain but highlights a stark disparity between the number of trials conducted for back pain in children and adolescents versus the extensive research conducted in adults. While there are some similarities in guideline recommendations for adults and young people [49], there remains a significant gap in the adaptation of guidelines and treatments for young people.

A strength of our analysis of COAST and O-COAST study data was the prospective nature of data collection. Unlike previous utilisation studies based on parent-reported surveys or population studies, the prospective observation of encounters minimise the likelihood of recall bias during data collection. The data analysed came from two similar but geographically separate populations. Chiropractic regulation differs structurally between the two countries: Australia regulates the profession nationally through the Australian Health Practitioner Regulation Agency (AHPRA) and the Chiropractic Board of Australia, while Canada regulates provincially, with each province maintaining its own regulatory college. We consider these structural differences unlikely to materially affect the RFE, diagnosis, and treatment patterns reported here, and have therefore pooled the two datasets. Previous reports show the sample of clinicians typically represented chiropractors from Victoria and Ontario [34, 35, 50]. Although participation in the primary COAST and O-COAST studies was voluntary, both studies found participating chiropractors to be broadly representative of the wider chiropractic workforce on key demographic and practice characteristics; however, the potential for selection bias cannot be completely excluded. Given our results closely mirror findings of studies in Europe and the United States, we suggest our study provides plausible, more detailed, insights into paediatric practice in the chiropractic profession. A limitation of this secondary analysis is that the data were collected more than a decade ago and may not reflect contemporary patterns of paediatric presentations to chiropractors. However, in the absence of more recent published evidence and in light of ongoing controversy surrounding chiropractic care for children in Australia [51, 52], re-examining patterns of service utilisation and the characteristics of paediatric encounters remains important to inform both the profession and the public. A further limitation of this secondary analysis relates to the measurement tools used in the primary studies. Questionnaires were not designed to comprehensively capture information specific to paediatric samples. Hence, our findings are broad and without nuanced detail pertinent to each age strata for this special population [21, 22]. Similarly, the type of manual therapy used, indication of modification for younger patients (under 18 years), or information regarding adverse events from treatment were not recorded in the COAST or O-COAST studies.

Future research is needed to build on the findings of our study including the psychosocial aspects of care, particularly parent-practitioner relationships and their impact on perceived outcomes. The prevalence of health maintenance as a reported RFE, necessitates further exploration of parents’ desire to be involved and proactive in their children’s healthcare and the potential role of chiropractic care in integrated paediatric healthcare models. Moreover, there is a critical need to distinguish between perceived benefits and clinical effectiveness of the care provided. While common RFEs such as colic, sleep disturbances, and musculoskeletal issues pose challenges for conventional medicine and frequently follow self-limiting courses or fluctuating clinical courses, with often unclear aetiology and no single proven treatment [4, 17, 53, 54], comparisons of the effectiveness of chiropractic and conventional paediatric care for these conditions are premature. Priority should be given to a comprehensive understanding of the clinical course, prognosis, and aetiology of these conditions in young people before developing targeted, evidence-based treatments. Once these foundational aspects, framed within an appropriate theoretical rationale, are better understood, research can then focus on evaluating the long-term outcomes and safety profile of chiropractic care for young people.

## Conclusion

Through a secondary analysis of the COAST and O-COAST data, we have described the characteristics of paediatric patients who seek chiropractic care and the nature of the encounter. Almost half of these encounters with young patients were adolescents; “health maintenance” was the most common RFE across all age strata. Back problems were consistently identified as the most frequent diagnosis in paediatric encounters by chiropractors, irrespective of age. This points to an incongruence between the reasons patients (or their families) sought care, most commonly health maintenance, and the problems chiropractors diagnosed, most commonly back problems. Manual adjustment was the predominant treatment modality, with its application increasing with patient age. Prospective research is needed to better understand the use and role of chiropractic service provision for young patients. Such investigations are essential for directing chiropractic healthcare resources and empowering families to make well-informed decisions regarding their children’s healthcare.

## Supplementary Information

Below is the link to the electronic supplementary material.

Supplementary Material 1

## Data Availability

The datasets used and/or analysed during the current study are available from the corresponding author on reasonable request

## Abbreviations

COAST: Chiropractic Observation and Analysis Study
HREC: Human research ethics committee
ICPC-2: International Classification of Primary Care
O-COAST: Ontario-Chiropractic Observation and Analysis Study
REB: Research ethics board
RFE: Reasons for Encounter
STROBE: Strengthening the Reporting of OBservational studies in Epidemiology
